# The Effect of Initial Texture on the Plastic Deformation of Gradient Aluminum

**DOI:** 10.3390/ma16072603

**Published:** 2023-03-24

**Authors:** Hao Lyu, Yaxin Zhang, Yuan Bao, Jiahui Zhang

**Affiliations:** 1 College of Transportation Engineering, Dalian Maritime University, Dalian 116026, China; z2095@dlmu.edu.cn (Y.Z.); 15235227541@163.com (J.Z.); 2 Information Science and Technology College, Dalian Maritime University, Dalian 116026, China

**Keywords:** gradient aluminum, dislocations, texture

## Abstract

The effect of specific processing-induced surface textures in gradient aluminum has not yet been investigated. A dislocation-based multi-scale framework is employed to explore the influence of various initial shearing textures and the depth from the surface of the region featuring each texture on the macroscopic behavior of gradient aluminum. By assigning different textures to the same grain size gradient aluminum sample, the initial texture was found to significantly affect the plastic deformation and macroscopic behavior of gradient aluminum. Specifically, the {111} texture can enhance the strength–ductility synergy, and this effect is dependent on the depth from the surface where the texture is located. This texture can lead to a slow stress/strain gradient in the assigned texture region and a sharp stress/strain gradient in the grain size gradient region connecting this region with the coarse grain region. Particularly, the sharp stress/strain gradient can result in extra strengthening by adjusting the stress/strain localization. These findings provide valuable insights for the design and optimization of surface textures in gradient aluminum.

## 1. Introduction

Over the past few years, there has been increasing attention toward the development of metals with gradient microstructures [1,2]. These gradient microstructures typically consist of a grain size gradient, with a transition from nanograins at the surface to coarse grains in the center. This microstructural feature can address the strength–ductility trade-off dilemma and lead to promising macroscopic behaviors [3]. Fang et al. [4] prepared gradient copper by surface mechanical grinding treatment (SMGT) and attributed the superior mechanical properties to the grain growth of nanograined Cu. Wei et al. [5] demonstrated that the formation of a gradient hierarchical nanotwinned structure is beneficial for both strength and tensile ductility. Wu et al. [6] reported that gradient structures generate characteristic extra strain hardening and that uniaxial stretching generates a strain gradient along the depth, which facilitates dislocation accumulation and interaction leading to enhanced ductility. Moering et al. [7] revealed that the sample was strengthened by the complex initial stress state of the material combined with dynamic strain hardening within the mismatched layers, thus producing a 3D complex stress state.

To attain such microstructures, substantial effort has been devoted to modifying severe plastic deformation processing methods, which allow for the development of unique grain structures and a subsequent increase in strength and ductility. Various severe plastic deformation methods such as surface mechanical attrition treatment (SMAT) [8], surface mechanical rolling treatment (SMRT) [9], surface mechanical grinding treatment SMGT [10,11], high-pressure torsion (HPT) [12], and equal-channel angular pressing (ECAP) [13,14] can be used to produce different types of gradient microstructures with distinct grain size distributions and grain size spatial distributions. However, relatively less attention has been paid to the inevitable induced texture or texture gradient that accompanies severe plastic deformation processing. Moering et al. [15] evaluated the texture distribution from the top surface to the center of the low-carbon steel sample with a gradient structure, finding that shear strain was strongly correlated with texture. Chen et al. [16] observed similar differences in the texture of heterogeneous structures in pure aluminum laminates. Wang et al. [17,18] demonstrated that rolling can induce a gradient in the grain direction and that texture affects the mechanical properties of gradient hot-rolled 1561 aluminum alloy. To unveil the effect of initial texture, a thorough understanding of the effects of different initial texture fibers on gradient materials is necessary.

Numerical study could be used to investigate the effect and evolution of specific textured fibers; however, this is limited by the large number of grains in such multi-scale microstructures. For example, the crystal plasticity finite element method (CPFEM)-based method can also predict the texture evolution and the deformation behavior of polycrystals with gradient grain size [19,20,21]. Considering large numbers of grains, the visco-plastic self-consistent (VPSC) model developed by Lebenson and Tome [22] enables the rapid prediction of the evolution of the texture of different deformation mechanisms. Currently, by combining a dislocation evolution law with the VPSC model, it is possible to capture the evolution of dislocation density [23,24,25,26]. Furthermore, in order to account for the grain size effect, an intrinsic length scale must be introduced into the model. The geometrically necessary dislocations can introduce an intrinsic length scale, which is responsible for accounting for lattice curvature and, in a manner of speaking, inhibiting the motion of mobile dislocations [27,28]. The plastic strain gradient is closely related to the GND density [29,30], and the GND content can be determined from the plastic strain gradient value. Foley et al. [31] found that GND generation varies greatly with lattice orientation and is also influenced by microstructural factors such as texture evolution and grain boundaries. Furthermore, stress gradients arising from dislocation pile-ups against grain boundaries under inhomogeneous stress can also lead to this intrinsic length scale, resulting in effects that depend on grain size [32,33,34]. A combined stress/strain gradient model [35,36] was also developed and used to investigate the grain size effect in gradient materials.

This work is, therefore, conducted to clarify the effect of initial textures with particular common shearing texture fibers on the plastic deformation of gradient aluminum by using a previously developed multi-scale framework [37] based on dislocation activities. Tensile tests were performed on samples containing different types of shear/roll textured fibers, the plastic deformation and overall behavior of which was compared to samples with randomly oriented grains. The effects of varying depths on plastic deformation due to strong textures are analyzed and discussed.

## 2. Materials and Methods

### 2.1. CDD-VPSC Model

The pre-proposed multi-scale framework [23,37] incorporates the combination of a continuum dislocation dynamics (CDD) model and a visco-plastic self-consistent model in order to analyze the grain size effects on materials with heterogeneous microstructures. In this framework, the VPSC model is primarily responsible for capturing the macroscopic behavior of a polycrystal represented by grains with orientations and volume fractions. Each grain is treated as an inclusion embedded in the viscoplastic medium [22]. Local deformation within a grain is governed by a dislocation density evolution law, which can be traced using the CDD model. The Orowan relation was utilized to bridge these two models by substituting the conventional power law equation into a dislocation density and velocity based relation, as follows:

(1) $${\dot{\gamma }}^{s}=\rho_{M}^{s}b{\bar{v}}_{g}^{s}$$

where ${\dot{\gamma }}^{s}$ is the shear rate of slip system s, which is induced by the motion of mobile dislocations with density $\rho_{M}^{s}$, the Burgers vector b, and average glide velocity ${\bar{v}}_{g}^{s}$. The conventional power law is implicitly included in the term ${\bar{v}}_{g}^{s}$, which can be written as follows:

(2) $${\bar{v}}_{g}^{s}=\left\{\begin{array}{ll} v_{0}{\left|\frac{\tau^{s}}{\tau_{\mathrm{CR}}^{s}}\right|}^{\frac{1}{r}}\mathrm{sign}\left(\tau^{s}\right) & \tau^{s}\geq \tau_{\mathrm{CR}}^{s}\\ 0 & \tau^{s}\leq \tau_{\mathrm{CR}}^{s} \end{array}\right.$$

in which $v_{0}$ is the reference velocity in the order of $10^{-4}$, r is the strain rate sensitivity. The activation of a slip is determined by the ratio of resolved shear stress $\tau^{s}$ over the critical resolved shear stress $\tau_{\mathrm{CR}}^{s}$. The resolved shear stress $\tau^{s}$ can be obtained by applying the Schmid law to slip system s. The term $\tau_{\mathrm{CR}}^{s}$ represents the minimum stress to move a dislocation, which can be decomposed as follows:

(3) $$\tau_{\mathrm{CR}}^{s}=\tau_{0}^{s}+\tau_{H}^{s}+\tau_{\mathrm{SG}}^{s}$$

where $\tau_{0}^{s}$ is the lattice friction, $\tau_{H}^{s}$ stands for the hardening resistance due to interaction between slips, $\tau_{\mathrm{SG}}^{s}$ is a grain-size-dependent term related to the grain boundary strengthening (see Appendix A). The hardening term $\tau_{H}^{s}$ can be expressed as the Bailey–Hirsch hardening [38]:

(4) $$\tau_{H}^{s}=\sum_{\beta =1}^{N}\Omega^{s\beta }c^{*}\mu b\sqrt{\rho_{T}^{\beta }}$$

where $\Omega^{s\beta }$ is an interaction matrix between slip s and slip β, which can be obtained from discrete dislocation dynamics (DDD) simulations [39]. $c^{*}$ is a constant, and μ is shear modulus. $\rho_{T}^{\beta }$ represents the total statistically stored dislocation density, which can be further decomposed into the two terms $\rho_{M}^{\beta }$ and $\rho_{I}^{\beta }$. The evolution equations for mobile and immobile dislocation density are based on different dislocation interaction mechanisms, which are as follows:

(5) $${\dot{\rho }}_{M}^{s}=q_{1}\rho_{M}^{s}\frac{{\bar{v}}_{g}^{s}}{{\tilde{l}}_{g}^{s}}-q_{2}2R_{c}{\left(\rho_{M}^{s}\right)}^{2}{\bar{v}}_{g}^{s}-q_{3}\rho_{M}^{s}\frac{{\bar{v}}_{g}^{s}}{{\tilde{l}}_{g}^{s}}+q_{4}{\left(\frac{\tau^{s}}{\tau_{\mathrm{CR}}^{s}}\right)}^{\frac{1}{r}}\rho_{I}^{s}\frac{{\bar{v}}_{g}^{s}}{{\tilde{l}}_{g}^{s}}+\;q_{5}\sum_{\beta =1}^{N}P^{s\beta }\rho_{M}^{s}\frac{{\bar{v}}_{g}^{s}}{{\tilde{l}}_{g}^{s}}-q_{6}R_{c}\rho_{M}^{s}\rho_{I}^{s}{\bar{v}}_{g}^{s}-q_{7}R_{c}^{3}\rho_{M}^{s}{\left(\rho_{I}^{s}\right)}^{2}{\bar{v}}_{g}^{s}\pm p{\bar{v}}_{g}^{s}\cdot \nabla \rho_{M}^{s}$$

(6) $${\dot{\rho }}_{I}^{\alpha }=q_{3}\rho_{M}^{s}\frac{{\bar{v}}_{g}^{s}}{{\tilde{l}}_{g}^{s}}-q_{4}{\left(\frac{\tau^{s}}{\tau_{\mathrm{CR}}^{s}}\right)}^{1/r}\rho_{I}^{s}\frac{{\bar{v}}_{g}^{s}}{{\tilde{l}}_{g}^{s}}-q_{6}R_{c}\rho_{M}^{s}\rho_{I}^{s}{\bar{v}}_{g}^{s}+q_{7}R_{c}^{3}\rho_{M}^{s}{\left(\rho_{I}^{s}\right)}^{2}{\bar{v}}_{g}^{s}$$

where the first term describes the propagation and multiplication of the mobile dislocations. The second term refers to the mutual annihilation of two mobile dilsocations with opposite signs. The third term denotes the immobilization of mobile dislocations (i.e., formation jogs and junctions). The fourth term stands for the mobilization of the immobile dislocations. Cross-slip is considered in the fifth term. The sixth term represents the annihilation of mobile and immobile dislocations, such as the formation of dislocation dipoles. $\pm p{\bar{v}}_{g}^{s}\cdot \nabla \;\rho_{M}^{s}$ represents the dislocation flux that is responsible for the dislocation motion along the direction of glide velocity (which can be approximated using Equation (A4) in Appendix A). $q_{1}\sim q_{7}$ are parameters related to different dislocation activities, which can be obtained by fitting DDD simulations [34] or experiments [40]. ${\tilde{l}}_{g}^{s}$ is the mean free path of a gliding dislocation, which can be expressed as follows:

(7) $${\tilde{l}}_{g}^{s}=\frac{c}{\sqrt{\sum w^{\beta s}\left(\rho_{T}^{\beta }+\left|\left|\rho_{\mathrm{GND}}^{\beta }\right|\right|\right)}}$$

in which c is a numerical factor in the order of 1. $w^{\beta s}$ is a matrix similar to the interaction matrix. The term $\left|\left|\rho_{\mathrm{GND}}^{\beta }\right|\right|$ is introduced here to encompass the intrinsic size effect, which is further discussed in Appendix A. $R_{c}$ is the critical interaction radius, p stands for the probability of triggering the slip transmission. $P^{s\beta }$ is an N by N probability matrix that describes the chance of a screw dislocation cross-slip from slip system s to β, the value of which can be determined through Monte Carlo simulations.

In the VPSC model developed by [22], the local deformation within each single crystal is as follows:

(8) $${\dot{\varepsilon }}_{ij}\left(\bar{x}\right)={\dot{\gamma }}_{0}\sum_{s=1}^{N}m_{kl}^{s}{\left(\frac{m_{kl}^{s}\sigma_{kl}\left(\bar{x}\right)}{\tau_{\mathrm{CR}}^{s}}\right)}^{1/r}$$

where $m_{kl}^{s}$ is the Schmid tensor, which satisfies $m_{kl}^{s}=\frac{1}{2}\left({\vec{m}}_{k}^{s}⊗{\vec{n}}_{l}^{s}+{\vec{n}}_{l}^{s}⊗{\vec{m}}_{k}^{s}\right)$, in which ${\vec{m}}^{s},{\vec{n}}^{s}$ are slip direction and slip plane normal direction on slip system s, which remain invariant in crystal axes. ${\dot{\varepsilon }}_{ij}\left(\bar{x}\right)$ and $\sigma_{kl}\left(\bar{x}\right)$ are local strain rate and stress, respectively. By linearizing Equation (8), it can be rewritten as follows:

(9) $${\dot{\varepsilon }}_{ij}\left(\bar{x}\right)=C_{ijkl}^{\left(g\right)}\sigma_{kl}\left(\bar{x}\right)+{\dot{\varepsilon }}_{ij}^{0\left(g\right)}$$

in which $C_{ijkl}^{\left(g\right)}$ is the visco-plastic compliance of grain g, and ${\dot{\varepsilon }}_{ij}^{0\left(g\right)}$ is a back-extrapolated term in grain g. The average strain rate experienced in each grain can be expressed as follows:

(10) $${\dot{\varepsilon }}_{ij}=C_{ijkl}\sigma_{kl}+{\dot{\varepsilon }}_{ij}^{0}$$

The macroscopic strain rate at the polycrystal level follows similarly:

(11) $${\dot{E}}_{ij}={\bar{C}}_{ijkl}\Sigma_{kl}+{\overset{˙}{E}}_{ij}^{0}$$

The interaction between grain-level behavior and macroscopic-level behavior is defined as follows [41]:

(12) $$\left({\dot{\varepsilon }}_{ij}-{\dot{E}}_{ij}\right)-{\dot{\varepsilon }}_{ij}^{*}={\bar{C}}_{ijkl}\left(\sigma_{kl}-\Sigma_{kl}\right)$$

Equations (8) and (12) can be solved with the affine self-consistent scheme using the solution from Eshelby’s equivalent inclusion method [42].

### 2.2. Implementation of the Model

Here, a Voronoi tessellation was utilized to represent the polycrystal as VPSC is a dimensionless method. Each Voronoi cell symbolizes a grain that contains the information such as stress/strain state from the VPSC model, dislocation density from the CDD model, and neighboring information from Voronoi tesselation. By incorporating the spatial location of each grain, the framework becomes capable of approximating the local stress/strain and the dislocation density gradient. This is achieved through the use of the moving least square method, which allows for the accurate interpolation of data points within a given region; more details can be found in [41]. 

### 2.3. Materials

**Figure 1:**
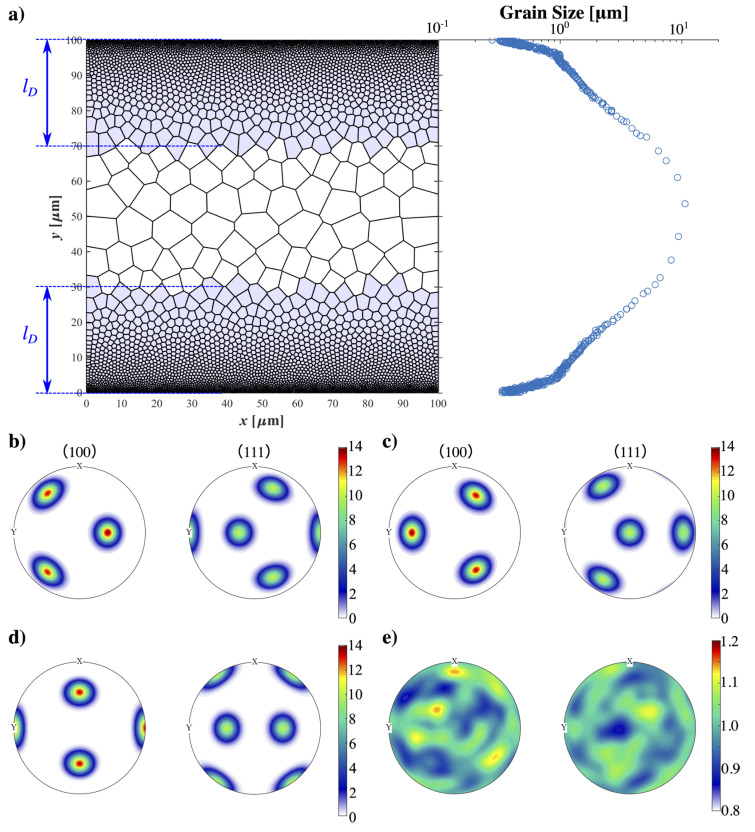
(**a**) Aluminum sample with graidient grain size along the y-direciton with assigned shearing texture (blue shading area) within $l_{D}$. Pole figures of assigned textures (**b**) A; (**c**) B; (**d**) C; and (**e**) random orientations.

In order to examine the effect of initial texture, various samples were generated on the same 2D Voronoi tessellation (100 µm × 100 µm) with a grain size gradient along the y-direction (see Figure 1), but with different texture fibers and different thicknesses of specific textures. We divided each sample into three regions: the nanograin region (NG) comprises two very thin layers on the surfaces with constant ultra-fine grain size (~200 nm); the coarse grain (CG) region is the large area located in the center with a grain size as large as 10 µm; the transit regions or the gradient grain size (GS) regions connect the NG and CG. Random grain orientations are assigned to grains in the CG region. 

The effect of initial texture fibers was investigated by assigning the respective textured fiber (see Table 1) into grains within a fixed depth ($l_{D}$) from the surface to the center. Then, samples were divided into three groups based on depth $l_{D}=10,\;20,\;30\;\mu m$, and in each group, 12 different texture fibers (adding ±5° noise to the Euler angles) were assigned to grains within the depths ($l_{D}$) from the surface to the center.

## 3. Results

In this work, tensile tests of gradient samples with various initial textures were performed along the y-direction under a strain rate of $10^{-3}$ s^−1^ with the same parameters used in [37] (see Table 2). The validation and determination of the parameters used for the VPSC-CDD model are also shown and discussed in [37]. The macroscopic behavior and dislocation density evolution of these samples were compared with that of a reference case with randomly assigned grain orientation.

**Figure 2:**
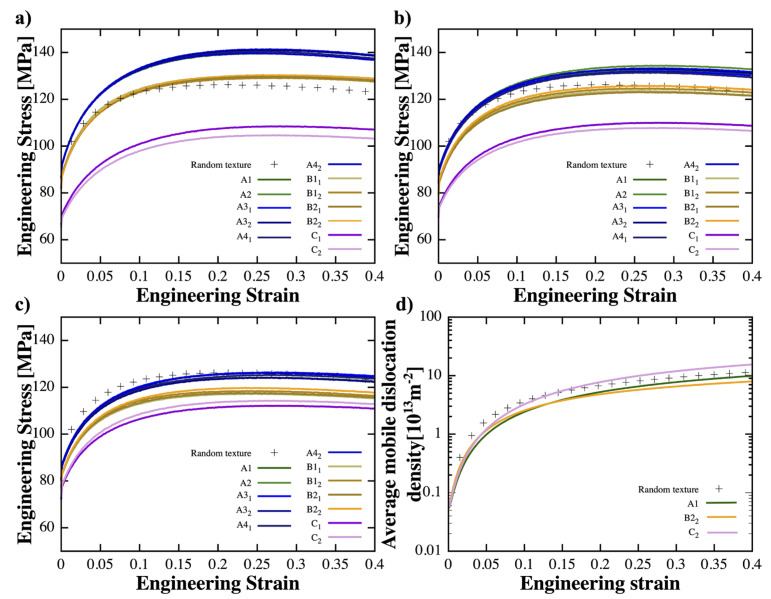
Stress–strain curves of different shearing textures within (**a**) $l_{D}=30\;\mu m$; (**b**) $l_{D}=20\;\mu m$; and (**c**) $l_{D}=10\;\mu m$. (**d**) Average mobile dislocation density vs. strain for three typical shearing textures.

For group I, different shearing textures were assigned to the grains in the NG and GS regions of the gradient sample with a depth of $l_{D}=30\;\mu m$. As shown in Figure 2a, the stress–strain curves of different textures can induce different macroscopic behavior. Texture {C} resulted in a lower yield strength of approximately 70 MPa, compared to the reference case. Texture {B} exhibited a similar trend of strain hardening at the initial strain stage and a later onset of instability ($\frac{d\sigma_{eng}}{d\epsilon_{eng}}=0$). Notably, the best combination of strength and ductility was achieved by inducing texture {A}. This texture led to a more significant strain hardening at an earlier strain stage and a tensile strength almost 1.2 times greater than the reference case. Though the onset of instability was found to occur earlier, the toughness of the cases with texture {A} was still the best among all the cases. When $l_{D}=20\;\mu m$ (group II), samples with texture {C} still displayed a similar trend with low strength. Interestingly, the initial strain hardening of samples with texture {A} and {B} was weakened, moving their stress–strain curves closer to that of the reference case. Even though the stress–strain curves of samples with texture {A} remained above the curve of the random texture, the tensile strength decreased to ~130 MPa. The curves of samples with texture {B} were almost entirely below the reference case. When the strong shearing texture was only present in the NG region ($l_{D}$ reaches 10 μm), all the shearing textures led to less strength than the reference case. These observations suggest that the initial texture can lead to significant differences in strength and the onset of instability. Texture {C} altered the advantage of gradient materials, resulting in a decrease in strength. Texture {A} contributed to more strengthening in comparison to other initial textures, and texture {B} could lead to better mechanical behavior depending on the $l_{D}$.

**Figure 3:**
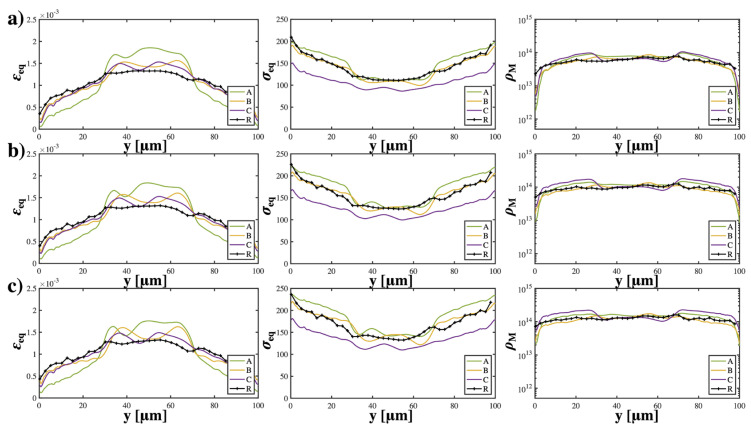
Spatial distribution of average equivalent strain ($\varepsilon_{eq}$), average equivalent stress ($\sigma_{eq}$ ), and (**c**) average mobile dislocation density $\rho_{M}$ vs. y for strain (**a**) 10%; (**b**) 20%; and (**c**) 30% when $l_{D}=30\;\mu m$.

**Figure A1:**
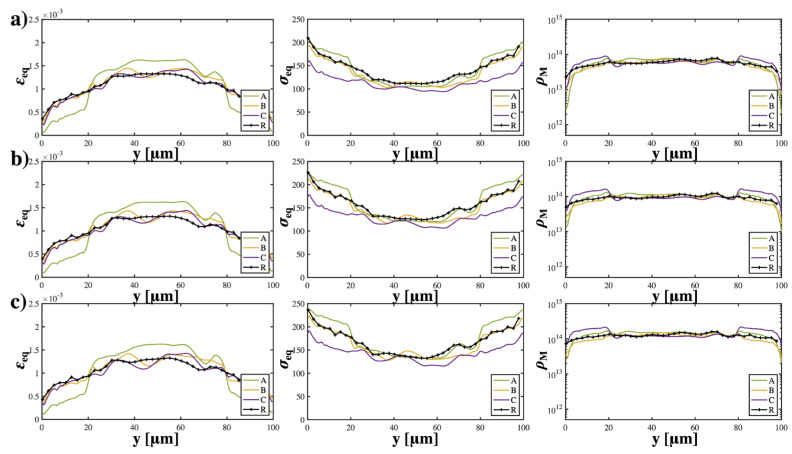
Spatial distribution of average equivalent strain ($\varepsilon_{eq}$), average equivalent stress ($\sigma_{eq}$ ), and (**c**) average mobile dislocation density $\rho_{M}$ vs. y for strain (**a**) 10%; (**b**) 20%; and (**c**) 30% when $l_{D}=20\;\mu m$.

**Figure A2:**
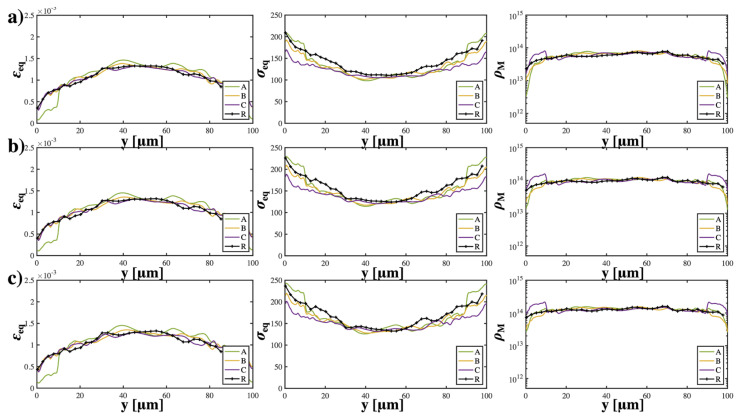
Spatial distribution of average equivalent strain ($\varepsilon_{eq}$), average equivalent stress ($\sigma_{eq}$ ), and (**c**) average mobile dislocation density $\rho_{M}$ vs. y for strain (**a**) 10%; (**b**) 20%; and (**c**) 30% when $l_{D}=10\;\mu m$.

**Figure A3:**
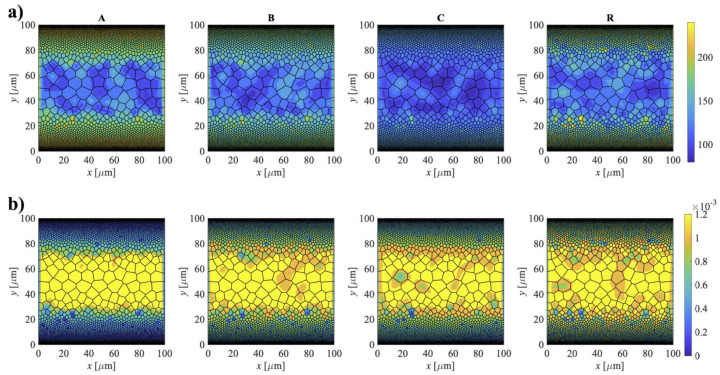
Contour plots of (**a**) equivalent stress and (**b**) equivalent strain for samples with different initial textures A, B, C, and random at 20% strain.

It is noteworthy that the average mobile dislocation density in cases with initial texture {C} was significantly higher than that of other cases, suggesting that the activation of slips requires overcoming a larger hardening barrier ($\tau_{H}$) induced by dislocation–dislocation interactions. This is reflected in the initial strain hardening rate and strength of cases with texture {C}, which were the lowest. This can be attributed to the local deformation caused by the vastly inhomogeneous microstructure. In addition, the stress gradient term plays an important role in determining the hardening rate, especially at the early strain stage, which is consistent with our previous work [44]. Figure 3 illustrates the average equivalent strain/stress and mobile dislocation density versus the grain size gradient direction Y at strain stages of 10%, 20%, and 30% for $l_{D}=30\;\mu m$. In all textures, grains in the CG region underwent greater plastic deformation than grains in the NG region, which barely deformed (see Figure A3 in Appendix B). Cases with texture {A} displayed a distinct equivalent strain/stress distribution along the y-direction, which demonstrates a log-like strain gradient from the surface to 30 μm followed by a stiffer strain gradient from 30 μm to the center. This is in contrast to the other textures, which displayed the opposite distribution trends. For cases with texture {A}, {B}, and random, the equivalent stress at the grains located at the NG region was significantly higher (approximately 200 MPa) than for cases with texture {C}, which was around 150 MPa. Texture {C} resulted in decreased strength and a relativiely slow stress gradient from the surface to the center along the y-direciton. The mobile dislocation density was significantly lower in grains at the NG region with texture {A} compared to texture {B}; however, the overall trends between these two textures remained quite similar. Intriguingly, texture {C} exhibited a distinct peak in mobile dislocation density at a depth of 30 μm. At different strain stages, the trends of stress and strain remained valid, though there was a slight upward shift in the curves. Regarding the trends of dislocation density, it was observed that all textures displayed an increase in mobile dislocation density at grains located in the NG region upon further loading; the contrast between grains located in the NG region and those in the center gradually diminished with increased loading. For samples with an $l_{D}$ of 20 μm (see Figure A1 in Appendix B), similar trends were observed; however, the stress/strain gradient began to change from the depth $l_{D}=20\;\mu m$ from the surface. For $\;l_{D}=10\;\mu m$, the sample with a randomly textured surface exhibited a strain gradient that was relatively similar to the other samples but a much higher stress gradient from a depth of 10 μm to the center along the y-direction (see Figure A2 in Appendix B). 

**Figure 4:**
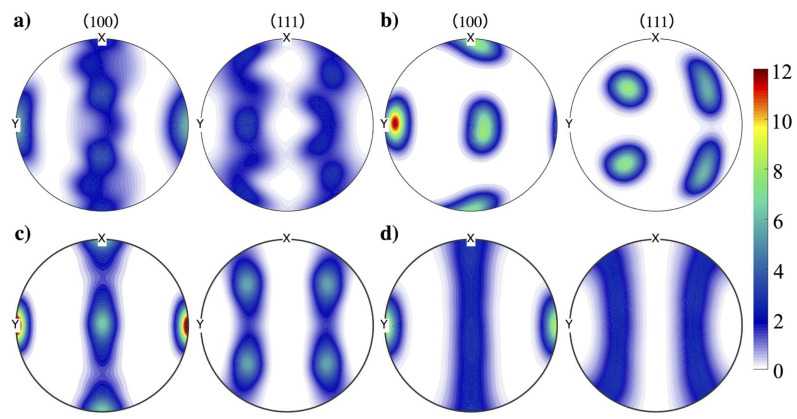
Pole figures of samples with assigned textures (**a**) A; (**b**) B; (**c**) C; and (**d**) random orientations within $l_{D}=30\;\mu m$ at 10% strain.

By plotting the pole figure of various initial textures at a strain of 10% (see Figure 4), we can observe that textures {B} and {C} exhibited a significant change in grain orientation from the original state to the loading direction. This suggests that more grains were aligned with the loading direction, leading to a faster increase in slips. In contrast, the intensity of grains aligned with the loading direction in samples with texture {A} was much weaker, at only around 1/6 of the intensity observed in other two textures. Despite the close similarity in contour between texture {A} and the random texture, the intensity of [010] orientation in the former was still significantly lower than that of the latter.

## 4. Discussion

Prior experimental investigations have shown the effect of gradient textures formed from the surface to the center on the strength and ductility on different alloys. Among them, Kuang et al. [45] found that gradient texture in an Al-Mn strip can improve the ductility of the alloy by inducing back stress, altering the local stress triaxiality, and delaying the fracture process. Moering et al. [7] also pointed out the effect of an out of plane {111} wire texture on the improvement of the mechanical behaivor of a gradient structured aluminum rod. In addition, they also found the existence of a shear texture gradient in SMATed low-carbon steel, which can contribute to the strength–ductility [15]. These studies reveal that the combination of gradient grain size and texture can create a synergistic effect on the strength and ductility of materials. While there have been reports on specific shearing texture and texture gradients in the NG and GS layer, less attention has been given to the synergetic effect of individual texture fibers and grain size gradients on the mechanical response. In this study, it was found that the {111} texture fiber can significantly enhance the strength of the material and delay the onset of instability when compared to samples with a random texture, regardless of the thickness of the layer featuring such texture. In Appendix C, Table A1 shows the Schmid factor for all slip systems of different textured fibers. The slips in {111} texture fiber show a significantly lower initial Schmid factor than the other groups, suggesting that fewer slips can be initiated. This fact is confirmed by the pole figure shown in Figure 4, which indicates that most grains can not be reoriented to align with the loading direction in the {111} texture at 10% strain. These grains located in the NG and GS region exhibit limited plastic deformation due to the size effect. The {111} texture effect leads to less resolved shear stress (RSS), making slip activation more difficult. Grains at the NG region display extremely low mobile dislocation density and small plastic strain, resulting in lower strain and stress gradients in comparison to the others within $l_{D}$. In grains located at the GS region, the mobile dislocation density can reach the order of $10^{14}$ $m^{-2}$. However, the grain size can limit the average gliding velocity of mobile dislocations, resulting in small strains. As the grain size varys with the y-direction, the plastic strain of the grains increases with y approaching 50 μm. Grains lying between $l_{D}$ and 50 μm have a random grain orientation. On one side, these grains are connected to {111}-textured grains in the GS region with a larger grain size and randomly distributed grain orientation, resulting in significantly larger strain and lower stress and leading to extremely high strain/stress gradients. Such huge stress/strain gradients can induce extra hardening and contribute to the strengthening of the samples. On the other side, these grains border the center coarse grains and show almost no difference in strain and stress. These grains can also act as a “thin wall” that isolates the stress localization area near the surface and the strain localization area in the center. Although there is still strain localization at the center and stress localization at the surface, the {111} texture can induce an expansion of the stress localization area and a reduction of the area of strain localization (see Figure A3 in Appendix B). The magnitude and the location of this strain/stress gradient varies with $l_{D}$, and vanishes when $l_{D}$ is approximately equal to the thickness of the NG region. Comparing our results with experimental work from Chen et al. [16], we found that the {110} and {112} textures can enhance the combination of strength and ductility by adjusting annealing time. Moreover, their results suggest that the {100}<110> texture induced by long time annealing can weaken the gradient aluminum, and this texture is usually accompanied by a larger grain size. Interestingly, Moering et al. [7] reported that the {111} texture developed after SMAT can contribute to strength and may lead to a different initial stress state of material and strain hardening, which was also our observation. This result unveils that the strength–ductility synergy of gradient aluminum could be further improved by introducing textures that can arouse a large strain/stress gradient in the GS region. Moreover, it should be emphasized that the thickness of the region characterized by the distinctive texture can significantly influence the macroscopic behavior of the material. Further investigations into the effects of gradient texture and specifically designed textures could lead to advancement in gradient materials.

## 5. Conclusions

In this work, a multi-scale dislocation-based framework is employed to investigate the synergetic effect of initial shearing texture and grain size gradient on aluminum. By varying the shearing texture and the area featuring respective strong shearing textures, we found that the initial shearing texture had a significant effect on the macroscopic behavior of gradient aluminum. The {111} texture can strengthen the gradient material and delay the onset of plastic deformation by causing a large stress/strain gradient near the border between the specific shearing texture and the random texture. This suggests the need for additional investigations into the effects of gradient textures and specific designed textures in gradient materials, as prior experiments have already shown the importance of gradient texture.

## Figures and Tables

**Table 1:** Common shearing textures for FCC metals [43].

Shear Texture	{hkl}<uvw>	Euler Angles (°)		
		$\varphi_{1}$	∅	$\varphi_{2}$
A_1_	$\left\{1\bar{1}\bar{1}\right\}$<110>	0	35.26	45
A_2_	$\left\{\bar{1}1\bar{1}\right\}$<$\bar{1}\bar{1}0$>	180	35.26	45
A_3_	$\left\{\bar{1}\bar{1}1\right\}$<112>	35.37	45	0
		125.37	90	0
A_4_	$\left\{11\bar{1}\right\}$<112>	144.74	45	0
		54.74	90	45
B_1_	$\left\{\bar{1}12\right\}$<110>	0	54.74	45
		120	54.47	45
B_2_	$\left\{1\bar{1}2\right\}$<$\bar{1}\bar{1}0$>	60	54.74	45
		180	54.74	45
C	{100}<110>	90	45	0
		0	90	45

**Table 2:** Parameters used in the simulation [37].

Symbol	Aluminum (Unit)
c* (Bailey–Hirsch hardening coefficient)	0.35
$\tau_{0}$ (internal friction)	4.5 MPa
$C_{11}$ (elasticity constant)	108.6 GPa
$C_{12}$ (elasticity constant)	61.3 GPa
$C_{44}$ (elasticity constant)	112 GPa
μ (shear modulus)	28.5 GPa
K (Hall–Petch constant)	0.047 MPa/mm^−1/2^
$v_{0}$(reference strain rate)	$1\times 10^{5}$ m/s
1/r (strain rate sensitivity)	0.05
b (magnitude of Burgers vector)	2.86 Å
$R_{C}$ (critical radius for annihilation coefficient)	15 b
$q_{1},\;q_{2},\;q_{3},\;q_{4},\;q_{5},\;\;q_{6},\;\;q_{7}$	0.0325, 2.0, 0.002, 0.002, 0.001, 0.002, 0.1
$\rho_{M}\;\rho_{\mathrm{IM}}$ (average mobile and immobile dislocation density)	$5\times 10^{11}$, $5\times 10^{11}$ $1/m^{2}$

## Data Availability

Not applicable.

## Appendix A

To capture the grain size effect, the intrinsic length scale was incorporated into the framework through GNDs and stress gradients. In Equation (7), the norm of the GND density $\left|\left|\rho_{GND}\right|\right|$ can be approximated by

(A1) $$\left|\left|\rho_{GND}\right|\right|=\frac{1}{b}\sqrt{AA}$$

where A is the Nyes dislocation tensor [27], the derivative of which can be approximated by

(A2) $$A=\mathrm{curl}\left(-F^{P}\right)$$

Here, $F^{P}$ is the plastic distortion, which can be obtained from the VPSC. The intrinsic length scale was introduced in the curl operator. In addition, another intrinsic length scale was incorporated in the size-dependent term $\tau_{\mathrm{SG}}$, which described the stress gradient aroused by dislocation pile-ups against the grain boundary under inhomogeneous shear stress. The simplified form of $\tau_{S}$ [33,44] can be expressed as

(A3) $$\tau_{\mathrm{SG}}=\frac{K}{\sqrt{L}}\left(1+\frac{L^{\prime }}{4\bar{\tau }}\left|\nabla \bar{\tau }\right|\right)$$

where L is the grain size, K is the Hall–Petch constant, $L^{\prime }$ is the average distance between obstacles (grain boundaries), which is treated as the grain size here. $\bar{\tau }$ is the equivalent stress, and the stress gradient term $\nabla \bar{\tau }$ brings in the intrinsic length scale. Note that when the system is under constant shear, this term vanishes and $\tau_{\mathrm{SG}}$ becomes the Hall–Petch relation. More details can be referred to in [36].

In addition, as discussed in Section 2.1, dislocation flux can occur when certain geometrical and stress conditions are met, resulting in the movement of dislocations across grain boundaries. In this work, we assume that the flux of dislocation is in the direction of a vector pointing from the center of one Voronoi cell to the center of its neighboring cell. Then, the flux term can be approximated by [46]

(A4) $$p{\bar{v}}_{g}^{s}\cdot \nabla \;\rho_{M}^{s}≅p{\bar{v}}_{g}^{s}\frac{\rho_{M}^{s\left(in\right)}-\rho_{M}^{s\left(out\right)}}{\bar{L}}$$

where $\bar{L}$ is the distance between the center of two grains, ${\bar{v}}_{g}^{s}$ is the average velocity of gliding dislocations, which is described in Equation (2). $\rho_{M}^{s}$ is the mobile dislocation density in slip system s, which can be evaluated by the evolution Equation (5). When slip transmission occurs between slip system s in grain A and slip system β in grain B, the dislocation on slip system s can travel through the grain boundary and enter grain B, which results in $\rho_{M}^{s}=\rho_{M}^{s\left(out\right)}$. Similarly, the dislocation on slip system β can also travel through the grain boundary and enter grain A, which results in $\rho_{M}^{\beta }=\rho_{M}^{s\left(in\right)}$. However, it is important to note that not all dislocations undergo slip transmission, so a slip transmission factor p, is introduced to account for this, which can be expressed as the equation proposed by Shi and Zirky [47], which is as follows:

(A5) $$p=\frac{M_{s\beta }^{\prime }\left(\tau_{out}^{\beta }/\tau_{C}^{\beta }\right)}{\sum_{i=1}^{N^{\prime }}(M_{si}^{\prime }\left(\tau_{out}^{i}/\tau_{C}^{i}\right)}$$

Here, $\tau_{out}^{\beta }$ is the resolved shear stress of the slip system β, where the outgoing dislocations on this slip systems can travel across the grain boundary by overcoming the critical resolved shear stress $\tau_{C}^{\beta }$. $M^{\prime }$ is a geometrical parameter between two neighboring grains, which can be expressed as follows:

(A6) $$M^{\prime }=\left({\vec{n}}_{in}\cdot {\vec{n}}_{out}\right)\left({\vec{m}}_{in}\cdot {\vec{m}}_{out}\right)$$

where ${\vec{n}}_{in}$ and ${\vec{n}}_{out}$ are the slip normal of incoming and outgoing slip systems, and ${\vec{m}}_{in}$ and ${\vec{m}}_{out}$ denote the slip direction of incoming and outgoing slip systems, respectively. The value of $M^{\prime }$ ranges from 0 to 1. Practically, when $M^{\prime }=0$, the chance for a dislocation to travel through the grain boundary becomes zero, and the grain boundary is impenetrable; when the value of $M^{\prime }$ is 1, dislocation can travel through the grain boundary once the driven stress is larger than the resistance. The resistance from the grain boundary will be used to replace $\tau_{\mathrm{SG}}$ in Equation (3), which is defined as follows:

(A7) $$\tau_{GB}=\left(1-M^{\prime }\right)\tau_{\mathrm{SG}}$$

Once the stress condition is satisfied, slip transmission can be triggered when the dislocation density of the outgoing slip systems is larger than that of incoming systems.

## Appendix C

**Table A1:** Schmid factor * for different slip systems for A_3_, B_1_, and C texture under tension along [010].

Slip System	A_3_	B_1_	C
[110]$(1\bar{1}$1)	−0.27	0	0
[011]$(1\bar{1}$1)	0	0	0.41
$[10\bar{1}$$](1\bar{1}$1)	−0.27	0	−0.41
[110]$(1\bar{1}\bar{1}$)	−0.27	0.47	0
[011]$(1\bar{1}\bar{1}$)	0	−0.15	−0.41
[101]$(1\bar{1}\bar{1}$)	0	0.11	−0.41
$[1\bar{1}$$0](11\bar{1}$)	0	0.37	0
[011]$(11\bar{1}$)	0	−0.21	−0.41
[101]$(11\bar{1}$)	0	0.15	−0.41
$[1\bar{1}$ 0](111)	0	0.11	0
$[0\bar{1}$ 1](111)	−0.27	0.15	0.41
$[10\bar{1}$ ](111)	0.27	0.26	−0.41

* The schimd factors were calculated using the MTEX tool box [48].
